# Seasonal variations in outcomes following spontaneous intracerebral hemorrhage: a case control study

**DOI:** 10.1186/s12883-026-04844-2

**Published:** 2026-03-26

**Authors:** Chenyi Zhan, Mengmeng Xu, Wenru Zhang, Chaomin Qiu, Hongyan Wang, Qianqian Chen, Kai Lin, Jingye Pan, Yunjun Yang, Dongqin Zhu

**Affiliations:** 1 Department of Radiology, The First Affiliated Hospital of Wenzhou Medical University, Wenzhou, China; 2 Department of Radiology, Taizhou Hospital of Zhejiang Province Affiliated to Wenzhou Medical University, Linhai, China; 3 Department of Nuclear Medicine, The First Affiliated Hospital of Wenzhou Medical University, Wenzhou, China; 4 Department of Intensive Care Unit, The First Affiliated Hospital of Wenzhou Medical University, Wenzhou, China

**Keywords:** Stroke, Intracerebral hemorrhage, Outcome, Seasonality

## Abstract

**Background:**

While seasonal variations in the incidence of spontaneous intracerebral hemorrhage (ICH) are well-recognized, the impact of onset season on outcomes remains poorly defined. This study investigated the association between the season of onset and outcomes in ICH patients.

**Methods:**

We retrospectively analyzed 363 consecutive ICH patients between May 2018 and May 2020 in the First Affiliated Hospital of Wenzhou Medical University. The primary outcome was poor 90-day functional recovery [modified Rankin Scale (mRS) 4–6]. Secondary outcomes included 90-day Barthel Index (BI) ≤ 60, prolonged length of stay (LOS), and hematoma expansion (HE). Univariate and multivariate logistic regression models were used to assess the relationship between onset season and outcomes. Subgroup analyses further evaluated the robustness of these findings.

**Results:**

Of the 363 patients included, 115 (31.7%) presented in spring, 54 (14.9%) in summer, 95 (26.2%) in autumn, and 99 (27.3%) in winter. Compared with winter, autumn onset was independently associated with a significantly lower risk of poor 90-day functional outcome (aOR^1^: 0.352, 95% CI: 0.186–0.666; aOR^2^: 0.394, 95% CI: 0.203–0.763). This protective effect remained consistent across subgroups, particularly in males, patients aged < 70 years, and those with a GCS score ≥ 5. In the GCS ≥ 5 subgroup, summer onset was associated with a lower risk of 90-day mRS 4–6 and BI ≤ 60. Autumn onset correlated with reduced risks of both BI ≤ 60 and HE, while spring onset was protective against HE. Additionally, winter onset was linked to the longest LOS.

**Conclusions:**

Winter onset was associated with the poorest functional outcomes and prolonged hospitalization. Conversely, autumn and summer onsets were protective against disability, while autumn and spring onsets were linked to a reduced risk of hematoma expansion. Further large-scale prospective validation is required to confirm these seasonal trends in ICH outcomes.

**Trial registration:**

The study has been registered at the Chinese Clinical Trial Registry (www.chictr.org.cn), number ChiCTR2200064360, registration date: October 4, 2022.

**Supplementary Information:**

The online version contains supplementary material available at 10.1186/s12883-026-04844-2.

## Background

Stroke was the third leading cause of death and disability globally in 2019, with a concerning upward trend in prevalence among the population under age 70 [1]. Although a winter peak in hemorrhagic stroke incidence has been established, the prognostic influence of seasonal onset remains poorly understood [2–6].

Spontaneous intracerebral hemorrhage (ICH) accounts for 10% to 15% of all strokes globally and is associated with a higher mortality and disability rate compared to other stroke subtypes [7–9]. Consequently, ICH exerts a significant socioeconomic burden [10], particularly in China [11], where the hospital length of stay (LOS) is a primary driver of inpatient costs [12, 13]. In addition, ICH is a dynamic pathology, hematoma expansion (HE) is a critical factor associated with subsequent neurological deterioration and death [14–16].

The primary objective was to evaluate the impact of seasonal onset on poor functional outcomes, defined as a 90-day modified Rankin Scale (mRS) score of 4–6. Secondary endpoints included 90-day Barthel Index (BI) scores, prolonged LOS, and the occurrence of HE.

## Methods

### Study design

We conducted a retrospective analysis of consecutive patients diagnosed with ICH at the First Affiliated Hospital of Wenzhou Medical University between May 2018 and May 2020. This tertiary referral center serves a catchment population of approximately 9.7 million in the subtropical region of southern Zhejiang Province. Patients were included in following criteria: 1) age ≥ 18 years, 2) primary spontaneous ICH diagnosed on non-contrast computed tomography (NCCT) within 6 h of symptom onset, with follow-up NCCT available within 72 h. Patients were excluded in following conditions: 1) primary isolated intraventricular hemorrhage (IVH), 2) secondary ICH due to vascular malformation, traumatic brain injury, or other intracranial pathologies, 3) ischemic stroke with hemorrhagic transformation, 4) surgical hematoma evacuation or any other neurosurgical intervention prior to follow-up NCCT. This study followed the Strengthening the Reporting of Observational Studies in Epidemiology (STROBE) guidelines for case–control studies. The study is registered with the Chinese Clinical Trial Registry (www.chictr.org.cn), with the registration number ChiCTR2200064360 (Registration Date:10/04/2022).

Consequently, patients were categorized into four groups based on the onset season: spring (March–May), summer (June–August), autumn (September–November), and winter (December–February).

### Baseline data and variables

Retrospectively reviewing the electronic medical record system, we recorded the following baseline data and variables: 1) demographic data (sex and age), 2) medical history (hypertension, diabetes mellitus, previous stroke history), 3) behavioral risk factors (alcohol consumption, smoking history), 4) initial clinical data (admission systolic blood pressure (SBP), diastolic blood pressure (DBP), Glasgow Coma Scale (GCS) score [17], time from symptom onset to CT), 5) imaging features (ICH location, baseline ICH volume, follow-up ICH volume, presence of IVH). The volumes of ICH and IVH were segmented and calculated using 3D Slicer software as in our previous studies [18, 19]. The previous stroke history included hemorrhagic and/or ischemic stroke. All clinical and imaging variables were collected by experienced investigators who were blinded to the outcomes of interest. Additionally, evaluations by trained radiologists validated the nontraumatic ICH diagnoses.

### Outcomes of interest

The primary outcome consisted of poor functional recovery at 90 days, categorized by a mRS score of 4–6 [14, 20, 21]. Secondary outcomes included the 90-day BI score ≤ 60 [22, 23], HE, and prolonged hospital LOS—the latter defined as > 14 days per established Chinese literature [11, 24]. HE was characterized by an increase of > 6 mL or > 33% in parenchymal volume, and/or a ventricular volume increase of ≥ 1 mL between the initial and follow-up CT scans [15]. The 90-day mRS and Bl score were obtained via telephone follow-up. LOS was extracted from the electronic medical record system.

### Statistical analysis

Categorical variables were expressed as counts (percentage) and compared using the Pearson chi-square test. Continuous variables were summarized as medians (with interquartile range, IQR) and compared using the Mann–Whitney test. Cases with missing data (5.51%) were excluded from the final analysis without imputation. Seasonal differences were analyzed via the Kruskal–Wallis H test, followed by Dunn-Bonferroni post-hoc pairwise comparisons where appropriate. We used univariate and multivariable logistic regression to calculate odds ratios (ORs) and 95% confidence intervals (CIs) for outcomes, designating winter as the reference category due to its established incidence peak [2, 3, 6]. Adjustments were made using two additive multivariable models [8, 16, 17, 25]. Model 1 accounted for pre-morbid factors, including age, sex, hypertension, diabetes, prior stroke, alcohol consumption, smoking. Model 2 addressed acute ICH severity by adjusting for age, sex, admission SBP/DBP, baseline ICH volume, and location.

Statistical analyses were conducted using R version 4.2.3 (R Foundation for Statistical Computing, Vienna, Austria). Statistical significance was set at *p* < 0.05, and all tests were two-tailed.

### Sensitivity analysis

Sensitivity analyses were conducted to further explore the prognostic effects of different onset seasons. Patients were stratified based on factors that may influence outcomes. The cohort was stratified by age (< 70 and ≥ 70 years) [1, 26–28] and sex. To assess the robustness of our findings, we further analyzed a subset of patients with GCS scores ≥ 5. This threshold was selected because a GCS of 5 serves as a pragmatic cutoff between aggressive neurosurgical intervention and palliative care [25, 29], particularly considering the relatively high surgical propensity for ICH patients in China [21, 26]. We also performed an additional subgroup analysis using a GCS score of 13 as the cutoff to represent patients with mild neurological impairment, a classification widely recognized in international clinical practice [17, 30].

## Results

### Comparison of baseline characteristics

A total of 363 patients were included in this study and categorized by their onset season. Of these, 115 (31.7%) had onset in spring, 54 (14.9%) in summer, 95 (26.2%) in autumn and 99 (27.3%) in winter. Baseline characteristics of ICH patients stratified by seasonal are presented in Table 1. Patients admitted in spring were significantly older (vs. summer and autumn), presented with lower DBP (vs. autumn), and exhibited shorter time from onset to CT (vs. winter; Supplementary Table 1). Furthermore, hospitalization cost was significantly lower in autumn than in winter (all adjusted *p* < 0.05). Although the Kruskal–Wallis test indicated significant intergroup variation in GCS categories across seasons (*p* = 0.005), subsequent Bonferroni-adjusted pairwise comparisons identified no specific significant differences, despite a marginal trend between autumn and winter (adjusted *p* = 0.058). No significant seasonal differences were found regarding sex, medical history, SBP, hematoma location, or baseline hematoma volume (all *p* > 0.05).

**Table 1:** Baseline Characteristics of ICH Patients Stratified by Seasonal

Characteristic	Spring (*N* = 115)	Summer (*N* = 54)	Autumn (*N* = 95)	Winter (*N* = 99)	*P* Value
Demographic					
Age in years, median (IQR)	63.0(54.0–73.0)	56.0(47.0–64.0)	55.0(47.0–67.0)	59.0(49.0–68.0)	0.007
Male, n (%)	83(72.2)	35(64.8)	67(70.5)	61(61.6)	0.350
Medical history^a^					
Hypertension, n (%)	88(80.7)	43(84.3)	73(81.1)	82(88.2)	0.483
Diabetes mellitus, n (%)	23(21.1)	13(25.5)	18(20.0)	19(20.4)	0.880
Previous stroke, n (%)	14(12.8)	8(15.7)	17(18.9)	16(17.2)	0.690
Smoking, n (%)	32(29.4)	8(15.7)	32(35.6)	27(29.0)	0.098
Alcohol consumption, n (%)	33(30.3)	9(17.6)	24(26.7)	25(26.9)	0.414
Admission measurements^a^					
SBP, mmHg, median (IQR)	155.0(143.0–168.0)	158.0(143.0–167.0)	161.0(144.0–171.0)	155.0(140.0–175.0)	0.449
DBP, mmHg, median (IQR)	87.0(80.0–96.0)	92.0(82.0–103.0)	94.0(83.0–102.0)	90.0(78.0–97.0)	0.010
GCS score, n (%)					0.005
13–15	66(60.6)	37(72.5)	68(75.6)	54(58.1)	
5–12	40(36.7)	8(15.7)	20(22.2)	32(34.4)	
3–4	3(2.8)	6(11.8)	2(2.2)	7(7.5)	
Imaging features					
ICH location, n (%)					0.888
Deep	90(78.3)	43(79.6)	76(80.0)	68(68.7)	
Lobe	7(6.1)	4(7.4)	6(6.3)	7(7.1)	
Brainstem	5(4.3)	3(5.6)	4(4.2)	9(9.1)	
Cerebellum	7(6.1)	2(3.7)	6(6.3)	9(9.1)	
Multiple locations	6(5.2)	2(3.7)	3(3.2)	6(6.1)	
Baseline ICH volume, ml, median (IQR)	14.4(7.1–26.2)	11.4(5.3–24.0)	14.0(6.3–25.7)	12.8(7.3–26.1)	0.716
Follow-up ICH volume, ml, median (IQR)	15.9(7.6–29.6)	12.7(6.7–23.8)	14.2(5.7–26.9)	15.1(8.4–32.6)	0.401
Presence of IVH, n (%)	42(36.5)	24(44.4)	37(38.9)	51(51.5)	0.136
Time from onset to CT, median (IQR)	2.5(2.0–3.5)	3.0(2.0–4.0)	2.5(1.5–4.0)	3.5(2.0–4.5)	0.030
Hospitalization cost^a^, RMB	28,553.0(17,300.0–63206.0)	29,291.0(15,402.0–60120.0)	25,882.0(15,746.0–49887.0)	42,475.0(21,956.0–73997.0)	0.021

*CT* Computed tomography, *DBP* Diastolic blood pressure, *GCS* Glasgow Coma Scale, *ICH* Intracerebral hemorrhage, *IQR* Interquartile range, *IVH* Intraventricular hemorrhage, *mRS* modified Rankin Scale, *RMB* Renminbi, *SBP* Systolic blood pressure

^a^Missing data for some variables (20/363, 5.51%)

Supplementary Table 2 details baseline characteristics by 90-day functional outcome. Relative to the favorable outcome group, patients with poor outcomes were older and more likely to have IVH or a history of stroke. The poor outcome group also displayed lower admission DBP, larger baseline and follow-up hematoma volumes, and higher hospitalization cost. Furthermore, GCS categories, hematoma location, and seasonal distribution differed significantly between groups (all *p* < 0.05).

### Comparison of outcomes

**Fig. 1:**
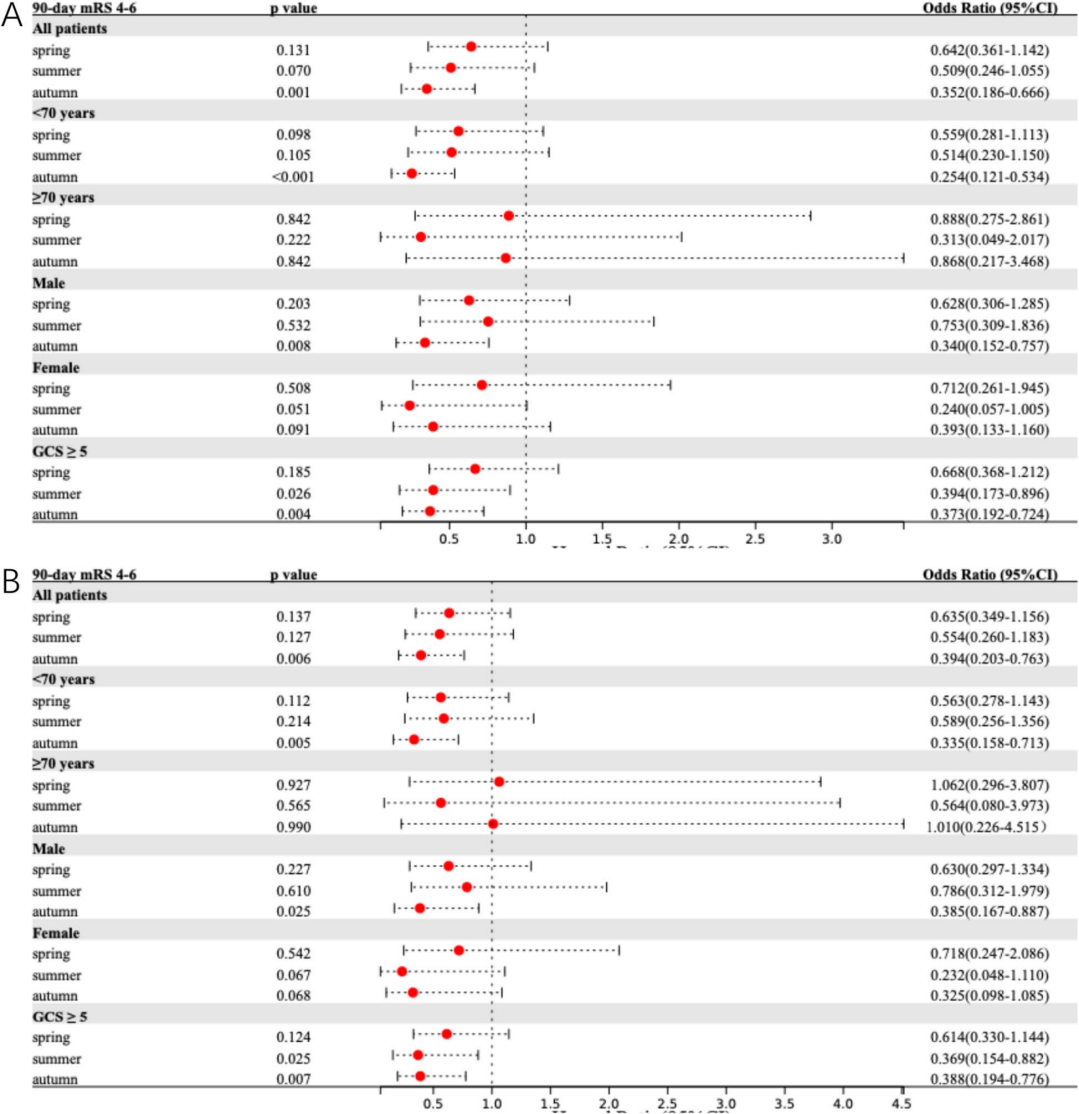
Comparison of 90-day mRS 4–6 among ICH patients by onset season. Multivariable-adjusted forest plot for 90-day mRS 4–6 in spontaneous ICH patients (reference: winter onset). Model 1 (**A**) adjusted for age, sex, hypertension, diabetes mellitus, previous stroke, smoking and alcohol consumption. Model 2 (**B**) adjusted for age, sex, systolic blood pressure, diastolic blood pressure, baseline ICH volume and ICH location. CI indicates confidence interval; GCS, Glasgow Coma Scale; ICH, intracerebral hemorrhage; mRS, modified Rankin Scale

**Fig. 2:**
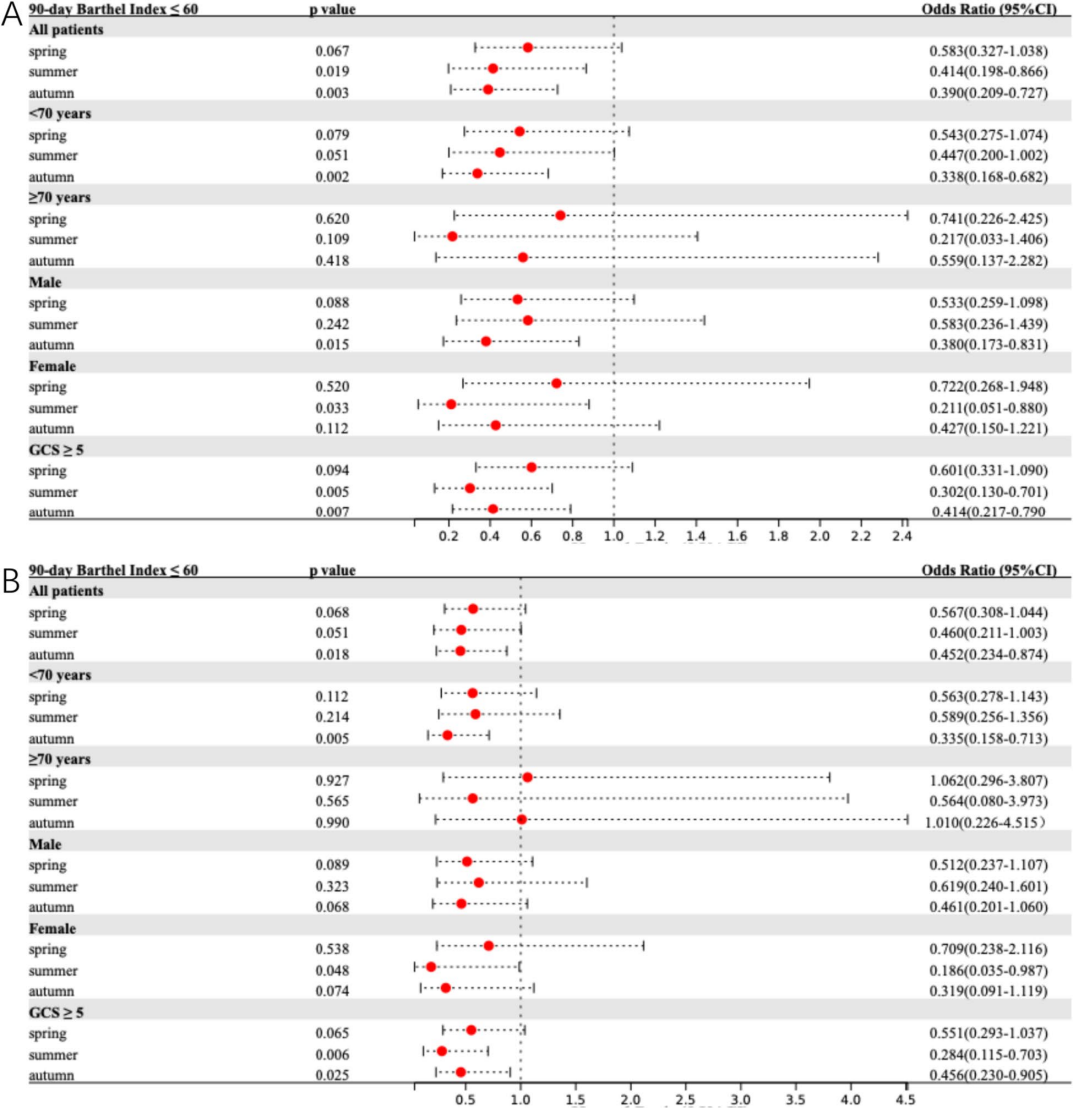
Comparison of 90-day Barthel Index ≤ 60 among ICH patients by onset season. Multivariable-adjusted forest plot for 90-day Barthel Index ≤ 60 in spontaneous ICH patients (reference: winter onset). Model 1 (**A**) adjusted for age, sex, hypertension, diabetes mellitus, previous stroke, smoking and alcohol consumption. Model 2 (**B**) adjusted for age, sex, systolic blood pressure, diastolic blood pressure, baseline ICH volume and ICH location. CI indicates confidence interval; GCS, Glasgow Coma Scale; ICH, intracerebral hemorrhage

**Fig. 3:**
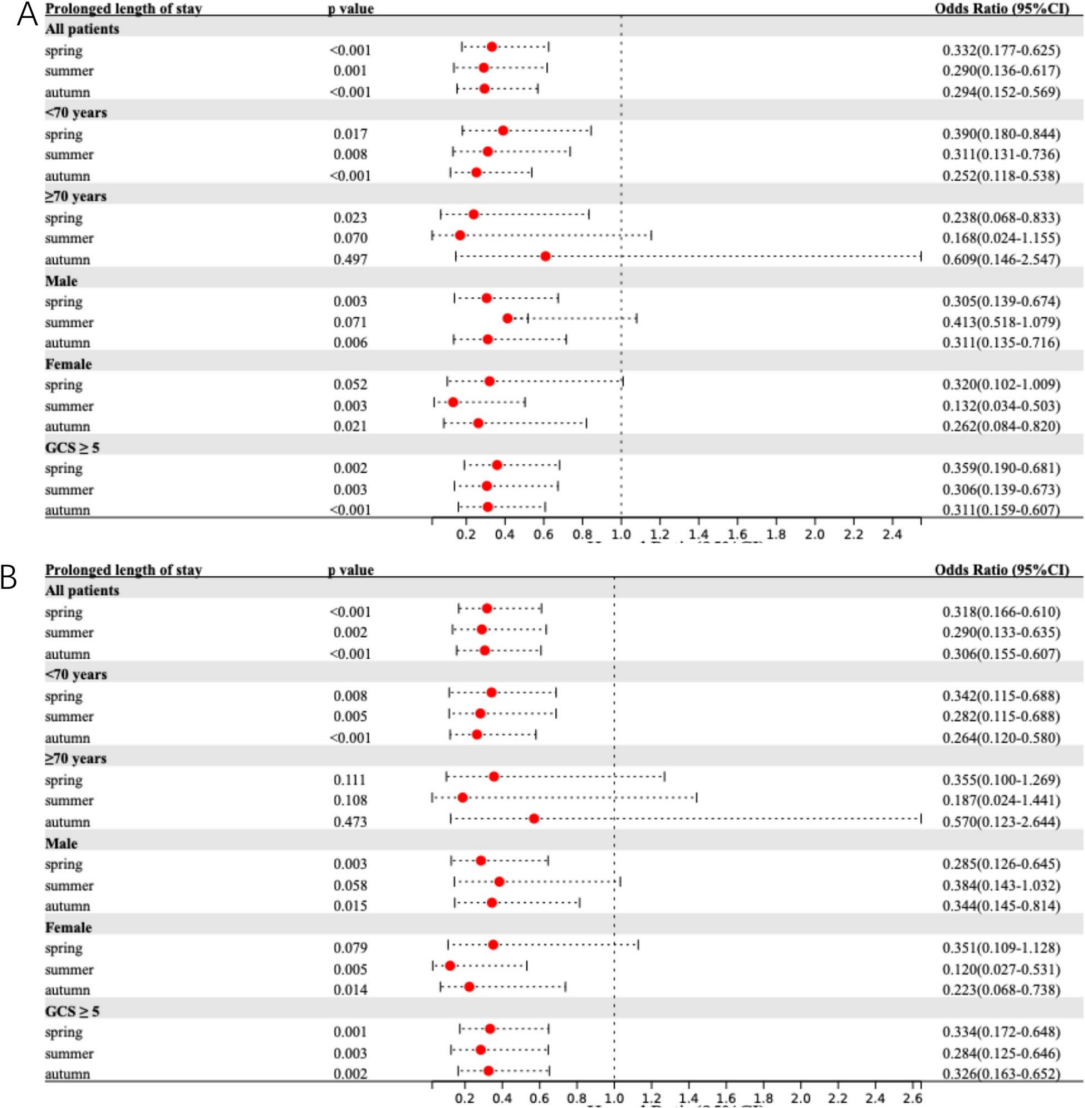
Comparison of prolonged length of stay among ICH patients by onset season. Multivariable-adjusted forest plot for 90-day prolonged length of stay in spontaneous ICH patients (reference: winter onset). Model 1 (**A**) adjusted for age, sex, hypertension, diabetes mellitus, previous stroke, smoking and alcohol consumption. Model 2 (**B**) adjusted for age, sex, systolic blood pressure, diastolic blood pressure, baseline ICH volume and ICH location. CI indicates confidence interval; GCS, Glasgow Coma Scale; ICH, intracerebral hemorrhage

**Fig. 4:**
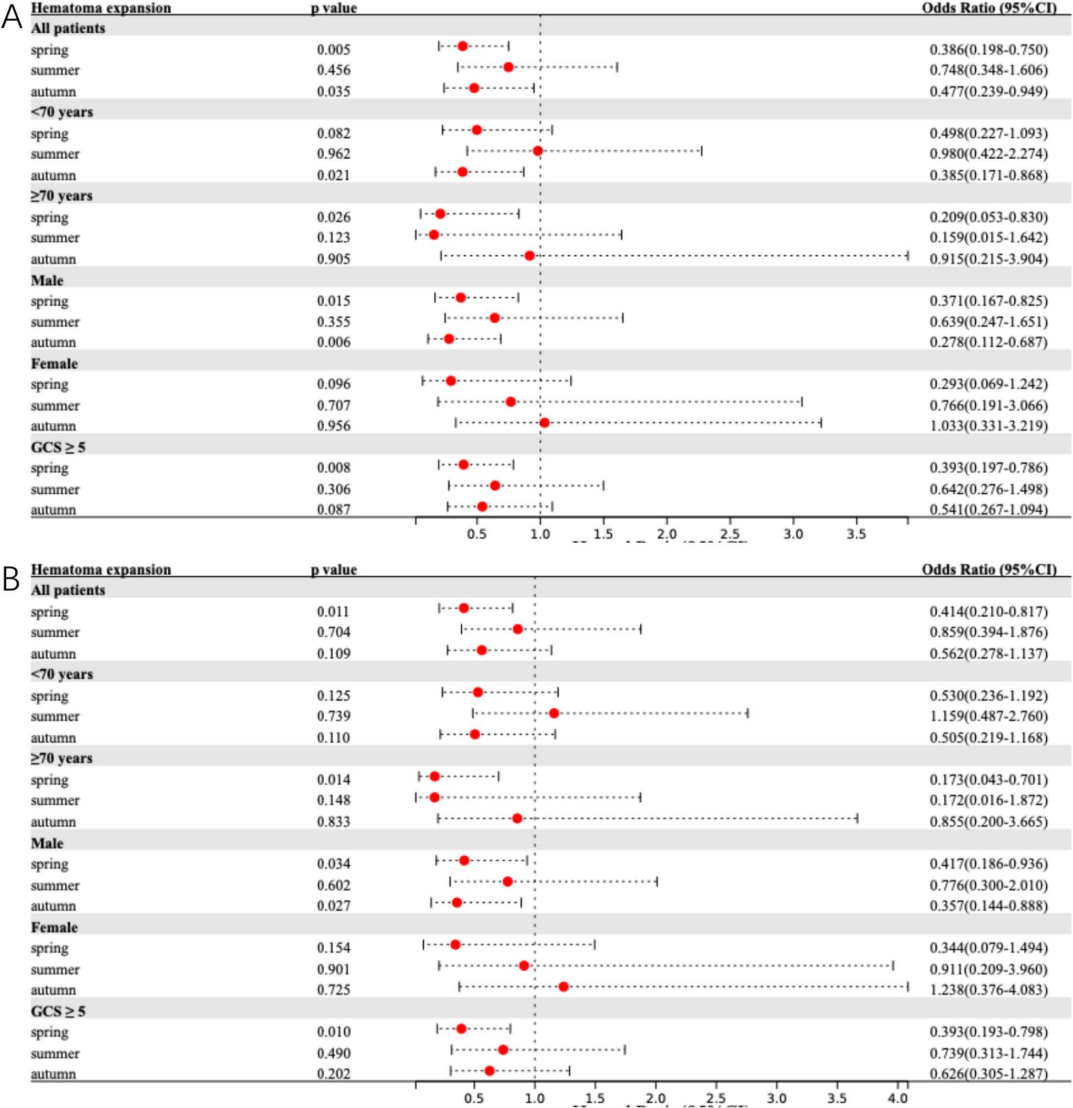
Comparison of hematoma expansion among ICH patients by onset season. Multivariable-adjusted forest plot for hematoma expansion in spontaneous ICH patients (reference: winter onset). Model 1 (**A**) adjusted for age, sex, hypertension, diabetes mellitus, previous stroke, smoking and alcohol consumption. Model 2 (**B**) adjusted for age, sex, systolic blood pressure, diastolic blood pressure, baseline ICH volume and ICH location. CI indicates confidence interval; GCS, Glasgow Coma Scale; ICH, intracerebral hemorrhage

Table 2 summarizes the seasonal variations in primary and secondary outcomes. Figure 1 presents adjusted models for the association between onset season and primary outcome across the total population and subgroups. Corresponding results for 90-day BI, prolonged LOS, and HE are detailed in Figs. 2, 3, and 4, respectively.

**Table 2:** Comparative analysis of primary and secondary outcomes for ICH patients by seasonal categories

**Outcome**	**Unadjusted OR (95% CI)**	*P* Value	**Adjusted OR**^**a**^** (95% CI)**	*P* Value	**Adjusted OR **^**b**^** (95% CI)**	*P* Value
90-day mRS 4–6						
Spring	0.671 (0.391–1.152)	0.148	0.642 (0.361–1.142)	0.131	0.635 (0.349–1.156)	0.137
Summer	0.491 (0.248–0.972)	0.041	0.509 (0.246–1.055)	0.070	0.554 (0.260–1.183)	0.127
Autumn	0.359 (0.198–0.651)	< 0.001	0.352 (0.186–0.666)	0.001	0.394 (0.203–0.763)	0.006
Winter	*Ref*	*Ref*	*Ref*	*Ref*	*Ref*	*Ref*
90-day Barthel Index ≤ 60						
Spring	0.615 (0.358–1.057)	0.079	0.583 (0.327–1.038)	0.067	0.567 (0.308–1.044)	0.068
Summer	0.400 (0.200–0.798)	0.009	0.414 (0.198–0.866)	0.019	0.460 (0.211–1.003)	0.051
Autumn	0.388 (0.216–0.695)	0.001	0.390 (0.209–0.727)	0.003	0.452 (0.234–0.874)	0.018
Winter	*Ref*	*Ref*	*Ref*	*Ref*	*Ref*	*Ref*
Prolonged length of stay						
Spring	0.320 (0.173–0.591)	< 0.001	0.332 (0.177–0.625)	< 0.001	0.318 (0.166–0.610)	< 0.001
Summer	0.303 (0.146–0.632)	0.001	0.290 (0.136–0.617)	0.001	0.290 (0.133–0.635)	0.002
Autumn	0.333 (0.176–0.631)	< 0.001	0.294 (0.152–0.569)	< 0.001	0.306 (0.155–0.607)	< 0.001
Winter	*Ref*	*Ref*	*Ref*	*Ref*	*Ref*	*Ref*
Hematoma expansion						
Spring	0.465 (0.255–0.850)	0.013	0.386 (0.198–0.750)	0.005	0.414 (0.210–0.817)	0.011
Summer	0.706 (0.346–1.438)	0.337	0.748 (0.348–1.606)	0.456	0.859 (0.394–1.876)	0.704
Autumn	0.476 (0.253–0.895)	0.021	0.477 (0.239–0.949)	0.035	0.562 (0.278–1.137)	0.109
Winter	*Ref*	*Ref*	*Ref*	*Ref*	*Ref*	*Ref*

*CI* Confidence interval, *ICH* intracerebral hemorrhage, *mRS* modified Rankin Scale, *OR* Odds ratio, *Ref.* Reference category

^a^Adjusted for age, sex, hypertension, diabetes mellitus, previous stroke, smoking and alcohol consumption

^b^Adjusted for age, sex, systolic blood pressure, diastolic blood pressure, baseline ICH volume and ICH location

Regarding the primary outcome, unadjusted analyses showed that summer and autumn onset were associated with better functional recovery compared to winter onset (OR 0.491, 95%CI 0.248–0.972 and OR 0.359, 95%CI 0.198–0.651, respectively). After adjusting for confounders, only autumn onset remained a significant independent predictor of favorable outcomes in both Model 1 (aOR^1^ 0.352, 95%CI 0.186–0.666) and Model 2 (aOR^2^ 0.394, 95%CI 0.203–0.763).

For secondary outcomes, unadjusted analyses indicated that summer and autumn onset were protective against a 90-day BI ≤ 60 (*p* < 0.05). In multivariate models, autumn onset consistently demonstrated a protective effect in Model 1 (aOR^1^ 0.390, 95%CI 0.209–0.727) and Model 2 (aOR^2^ 0.452, 95%CI 0.234–0.874), while summer onset was significant only in Model 1 (aOR^1^ 0.414, 95%CI 0.198–0.866). Notably, spring, summer, and autumn onset were all associated with a lower risk of prolonged LOS in both unadjusted and adjusted analyses (all *p* < 0.05), details in Table 2. Regarding HE, spring and autumn onset showed protective effects in unadjusted analyses (*p* < 0.05). Following adjustment, spring onset remained significantly associated with reduced HE risk in Model 1 (aOR^1^ 0.386, 95%CI 0.198–0.750) and Model 2 (aOR^2^ 0.414, 95%CI 0.210–0.817) compared to winter onset, whereas autumn onset reached significance only in Model 1 (aOR^1^ 0.477, 95%CI 0.239–0.949).

### Sensitivity analyses

Age-stratified sensitivity analyses are detailed in Supplementary Table 3. For the primary outcome, autumn onset (reference: winter) was a significant protective factor in patients < 70 years across the unadjusted model (OR 0.273, 95%CI 0.135–0.552), Model 1 (aOR^1^ 0.254, 95%CI 0.121–0.534) and Model 2 (aOR^2^ 0.335, 95%CI 0.158–0.713), whereas no significant association was found in those ≥ 70 years. Similarly, for a 90-day BI ≤ 60, autumn onset was protective only in the < 70 years group (unadjusted OR 0.344, 95%CI 0.175–0.675; aOR^1^ 0.338, 95%CI 0.168–0.682; aOR^2^ 0.465, 95%CI 0.223–0.972). Regarding prolonged LOS, spring, summer, and autumn onsets all served as protective factors for patients < 70 years (all *p* < 0.05, Supplementary Table 3). In contrast, only spring onset was protective in patients ≥ 70 years (unadjusted OR 0.258, 95%CI 0.082–0.815; aOR^1^ 0.238, 95%CI 0.068–0.833). For HE, autumn onset was protective in patients < 70 years (unadjusted OR 0.421, 95%CI 0.196–0.905; aOR^1^ 0.385, 95%CI 0.171–0.868), while spring onset showed a protective effect in patients ≥ 70 years across all models (unadjusted OR 0.237, 95%CI 0.077–0.731; aOR^1^ 0.209, 95%CI 0.053–0.830; aOR^2^ 0.173, 95%CI 0.043–0.701).

Sex-stratified sensitivity analyses are detailed in Supplementary Table 4. For the primary outcome, autumn onset (reference: winter) was consistently associated with a lower risk in male patients (unadjusted OR 0.359, 95%CI 0.173–0.745; aOR^1^ 0.340, 95%CI 0.152–0.757; aOR^2^ 0.385, 95%CI 0.167–0.887). In female patients, summer onset was a significant protective factor only in the unadjusted model (OR 0.240, 95%CI 0.067–0.858).

Regarding a 90-day BI ≤ 60, autumn onset was protective in males (unadjusted and Model 1), while summer onset showed significant protective effects in females across both unadjusted and adjusted models (all *p* < 0.05). For prolonged LOS, spring and autumn onsets were protective factors in males, while summer and autumn onsets were significant in females in both unadjusted and adjusted analyses (all *p* < 0.05). For HE, spring (Model 1 and 2) and autumn (all models) onsets were protective in males, whereas spring onset in females was significant only in the unadjusted model (*p* < 0.05).

Sensitivity analyses for ICH patients with GCS ≥ 5 are summarized in Supplementary Table 5. Regarding the primary outcome, summer and autumn onset were consistently identified as significant protective factors compared to winter onset in the unadjusted model (OR 0.372, 95%CI 0.167–0.829; OR 0.360, 95%CI 0.189–0.689), Model 1 (aOR^1^ 0.394, 95%CI 0.173–0.896; aOR^1^ 0.373, 95%CI 0.192–0.724) and Model 2 (aOR^2^ 0.369, 95%CI 0.154–0.882; aOR^2^ 0.388, 95%CI 0.194–0.776). Similarly, summer and autumn onset remained significant protective factors for a 90-day BI ≤ 60 across all models (*p* < 0.05). Spring, summer, and autumn onset were associated with a reduced risk of prolonged LOS, while spring onset specifically served as a protective factor against HE (all *p* < 0.05). Sensitivity analyses for ICH patients with GCS ≥ 13 are detailed in Supplementary Table 6.

## Discussion

Our findings indicated the significant influence of seasonal variation on ICH outcomes. Our primary analysis identified autumn onset as an independent protective factor against poor 90-day functional outcomes (mRS 4–6) compared to winter onset. This protective effect remained robust across key subgroups, including patients aged < 70 years, males, and those with GCS scores ≥ 5. Notably, summer onset also conferred a protective advantage for 90-day mRS within the GCS ≥ 5 subgroup.

Parallel to the primary outcome, autumn onset was associated with a reduced risk of functional dependence (90-dayBI ≤ 60), particularly in patients < 70 years and those with GCS ≥ 5. Regarding prolonged LOS, winter onset was linked to the longest LOS, whereas spring, summer, and autumn onset all showed relative advantages. Furthermore, spring onset appeared protective against HE in elderly patients and the GCS ≥ 5 subgroup, while autumn onset offered similar benefits for males and younger patients (< 70 years).

The impact of seasonality on ICH outcomes is not yet fully characterized. While seasonal variations in morbidity and mortality are extensively documented across various neurological and cardiovascular diseases [2, 31–35], previous research on ICH has primarily focused on incidence rather than outcomes. These studies consistently identified a peak in ICH incidence during the winter months [2, 3, 5, 36, 37].

The precise mechanisms underlying the seasonal influence on ICH outcomes remain poorly understood. Given its retrospective observational design, this study cannot establish a definitive causal relationship between seasonal variation and ICH outcomes. Nevertheless, the relative protective effects observed during spring, summer, and autumn compared to the winter peak may be driven by fluctuations in cardiovascular factors and physical activity levels. Previous studies have documented seasonal blood pressure variation, with elevated levels during winter cold exposure potentially contributing to higher ICH mortality [38–42]. Another potential mechanism involves cold-induced activation of the sympathetic and renin-angiotensin systems [41, 43, 44]. Given that hypertension is the primary risk factor for ICH incidence and poor prognosis [16, 45], these seasonal shifts are critical. Furthermore, conventional cardiovascular risk factors, including fibrinogen, triglycerides, and cholesterol levels, also exhibit distinct winter peaks [42, 46].

Our analysis revealed that winter onset was associated with the longest LOS. This trend is likely mediated by the increased incidence of cardiovascular and respiratory complications during winter [42, 47, 48]. Consistently, other acute conditions—including heart failure, myocardial infarction, and pulmonary embolism—exhibit a marked winter seasonality [48]. Moreover, the higher prevalence of respiratory infections during cold periods can trigger systemic inflammation and exacerbate underlying cardiovascular instability [47, 49]. Consequently, patients admitted in winter often present with a greater burden of multifactorial comorbidities. This heightened clinical complexity likely accounts for the prolonged LOS observed in winter, whereas spring, summer, and autumn are associated with significantly shorter hospitalizations.

Notably, our analysis identified autumn as a protective factor against poor 90-day functional outcomes. This may be attributed to the more moderate temperatures during autumn, particularly given that global warming has intensified summer heatwaves and winter cold extremes [50]. A prior study in China reported that 18.10% of hemorrhagic stroke mortality is attributable to non-optimal temperatures, especially in subtropical monsoon climates [51]. Furthermore, temperature fluctuations are known to exacerbate blood pressure levels during winter in hypertensive patients [38]. Given that over 80% of our cohort had a history of hypertension, such seasonal thermal variations likely influenced the observed protective effect. In contrast, a study in northern Israel (Mediterranean climate) found peak mortality in January and the lowest in February, while other functional outcomes remained statistically non-significant [52].

Our findings indicate that for males and patients aged < 70 years, autumn onset was a protective factor against poor 90-day functional outcomes (mRS 4–6) compared to winter. This subgroup-specific vulnerability may stem from increased exposure to cold stress among younger male individuals, potentially due to occupational demands [53]. Furthermore, seasonal variations in physical activity [54] —with younger males being more likely to engage in outdoor exercise—may exacerbate environmental exposure in winter. Dietary shifts also represent a plausible mechanism; higher intake of fats and cholesterol has been documented during winter months, particularly among patients in their 40 s and 50 s [55, 56]. Finally, the impact of air pollution, a recognized stroke risk factor [57], cannot be overlooked. Guo et al. [58] demonstrated a significant correlation between winter air pollution and ICH morbidity, especially in individuals aged ≥ 40 years, which aligns with the higher risk observed in our winter cohort.

The advantage of our study lies in relatively comprehensive assessment of the association between seasonal variation and ICH outcomes. In addition, multiple baseline characteristics were considered for adjusting potential confounders. Sensitivity analyses were also conducted to further explore the prognostic effects of different onset seasons.

Nonetheless, this study has several limitations. First, the retrospective and case–control design precludes the establishment of direct causality between seasonal onset and ICH outcomes, potentially increasing the risk of residual confounding. Furthermore, despite rigorous data extraction from electronic medical records by trained investigators, some clinical information may not have been fully captured. Second, due to the frequent under-recording of longitudinal functional outcomes in routine electronic medical records, 90-day mRS and BI scores were primarily collected via telephone interviews. Although subject to potential recall bias, this pragmatic approach ensured a more comprehensive follow-up, aligning with real-world clinical practice. Third, the relatively small sample size from a single center may limit the generalizability of our findings, particularly in tropical and frigid zone, and the statistical power of certain subgroup analyses. Future multi-center studies with larger cohorts are warranted to validate these results. Meanwhile, our study lacked data of temperature as temperature-based categorization. But our single-center design’s high homogeneity indicates significant reference value for ICH patients in temperate regions.

## Conclusion

Our findings suggest that seasonality significantly influences ICH outcomes. Compared to winter, autumn onset was independently associated with a reduced risk of poor 90-day functional outcomes (mRS 4–6 and BI ≤ 60), a trend consistently observed in males, patients aged < 70 years, and those with a GCS score ≥ 5. Summer onset also emerged as a protective factor for 90-day outcomes within the GCS ≥ 5 subgroup. Additionally, non-winter admissions were significantly associated with a shorter LOS. Spring and autumn onset were independently associated with a reduced incidence of early HE. Large-scale prospective studies are warranted to clarify the seasonal impact on ICH outcomes.

## Supplementary Information

Supplementary Material 1.

Supplementary Material 2.

## Data Availability

The data that support the findings of this study are available from the corresponding author upon reasonable request.
