# Supplementary material for: Seasonal variations in outcomes following spontaneous intracerebral hemorrhage: a case control study
Source: BMC Neurol. 2026 Mar 26;26:300. doi: 10.1186/s12883-026-04844-2 (PMC13141612; doi:10.1186/s12883-026-04844-2)
Supplement: Supplementary file 2 [file 12883_2026_4844_MOESM2_ESM.docx]

Supplementary Table1. Post-hoc Pairwise Comparisons of Characteristics Between Seasons

| Characteristic / Comparison | p Value a | p Value b |
| --- | --- | --- |
| Age in years |  |  |
| Spring vs. Summer | 0.005 | 0.031 |
| Spring vs. Autumn | 0.003 | 0.020 |
| Spring vs. Winter | 0.151 | 0.908 |
| Summer vs. Autumn | 0.748 | 1.000 |
| Summer vs. Winter | 0.118 | 0.709 |
| Autumn vs. Winter | 0.145 | 0.868 |
| DBP |  |  |
| Spring vs. Summer | 0.065 | 0.392 |
| Spring vs. Autumn | 0.002 | 0.012 |
| Spring vs. Winter | 0.541 | 1.000 |
| Summer vs. Autumn | 0.471 | 1.000 |
| Summer vs. Winter | 0.194 | 1.000 |
| Autumn vs. Winter | 0.107 | 0.102 |
| GCS score |  |  |
| Spring vs. Summer | 0.311 | 1.000 |
| Spring vs. Autumn | 0.036 | 0.214 |
| Spring vs. Winter | 0.556 | 1.000 |
| Summer vs. Autumn | 0.467 | 1.000 |
| Summer vs. Winter | 0.144 | 0.861 |
| Autumn vs. Winter | 0.010 | 0.058 |
| Time from onset to CT |  |  |
| Spring vs. Summer | 0.267 | 1.000 |
| Spring vs. Autumn | 0.551 | 1.000 |
| Spring vs. Winter | 0.004 | 0.025 |
| Summer vs. Autumn | 0.556 | 1.000 |
| Summer vs. Winter | 0.213 | 1.000 |
| Autumn vs. Winter | 0.030 | 0.183 |
| Hospitalization cost |  |  |
| Spring vs. Summer | 0.736 | 1.000 |
| Spring vs. Autumn | 0.242 | 1.000 |
| Spring vs. Winter | 0.048 | 0.288 |
| Summer vs. Autumn | 0.533 | 1.000 |
| Summer vs. Winter | 0.054 | 0.321 |
| Autumn vs. Winter | 0.003 | 0.015 |

Abbreviations: CT, computed tomography; DBP, diastolic blood pressure; GCS, Glasgow Coma Scale.

a raw p-value; b Bonferroni-adjusted p-value.

Supplementary Table2. Baseline Characteristics of ICH Patients Stratified by 90-day Functional Outcome

| Characteristic | Favorable outcome (mRS 0–3, N=216) | Poor outcome (mRS 4–6, N=147) | P Value |
| --- | --- | --- | --- |
| Demographics |  |  |  |
| Age, years, median (IQR) | 56.0 (49.0–67.0) | 61.0 (49.5–73.0) | 0.012 |
| Male, n (%) | 144 (66.7) | 102 (69.4) | 0.586 |
| Medical history a |  |  |  |
| Hypertension, n (%) | 178 (83.2) | 108 (83.7) | 0.896 |
| Diabetes mellitus, n (%) | 46 (21.5) | 27 (20.9) | 0.901 |
| Previous stroke, n (%) | 27 (12.6) | 28 (21.7) | 0.026 |
| Smoking, n (%) | 65 (30.4) | 34 (26.4) | 0.426 |
| Alcohol consumption, n (%) | 57 (26.6) | 34 (26.4) | 0.955 |
| Admission measurements a |  |  |  |
| SBP, mmHg, median (IQR) | 158.0 (144.0–169.0) | 157.0 (140.0–171.0) | 0.440 |
| DBP, mmHg, median (IQR) | 92.0 (82.0–100.0) | 88.0 (75.0–96.0) | 0.008 |
| GCS score, n (%) |  |  | <0.001 |
| 13–15 | 178 (83.2) | 47 (36.4) |  |
| 5–12 | 35 (16.4) | 65 (50.4) |  |
| 3–4 | 1 (0.5) | 17 (13.2) |  |
| Imaging features |  |  |  |
| ICH location, n (%) |  |  | 0.005 |
| Deep | 171 (79.2) | 106 (72.1) |  |
| Lobar | 14 (6.5) | 10 (6.8) |  |
| Brainstem | 11 (5.1) | 10 (6.8) |  |
| Cerebellum | 17 (7.9) | 7 (4.8) |  |
| Baseline ICH volume, mL, median (IQR) | 10.1 (5.8–19.7) | 18.4 (10.2–30.4) | <0.001 |
| Follow-up ICH volume, mL, median (IQR) | 9.7 (5.5–19.9) | 22.5 (11.7–40.0) | <0.001 |
| Presence of IVH, n (%) | 74 (34.3) | 80 (54.4) | <0.001 |
| Time from onset to CT, h, median (IQR) | 3.0 (2.0–4.0) | 3.0 (2.0–4.0) | 0.435 |
| Follow-up CT, h, median (IQR) | 23.8 (15.0–37.0) | 20.0 (11.8–29.0) | <0.001 |
| Season of onset, n (%) |  |  | 0.006 |
| Spring | 66 (30.6) | 49 (33.3) |  |
| Summer | 35 (16.2) | 19 (12.9) |  |
| Autumn | 68 (31.5) | 27 (18.4) |  |
| Winter | 47 (21.8) | 52 (35.4) |  |
| Hospitalization cost, RMB a, median (IQR) | 21574.0 (14513.0–39018.0) | 61118.0(28759.0–108898.0) | <0.001 |

Abbreviations: CT, computed tomography; DBP, diastolic blood pressure; GCS, Glasgow Coma Scale; ICH, intracerebral hemorrhage; IQR, interquartile range; IVH, intraventricular hemorrhage; mRS, modified Rankin Scale; RMB, Renminbi; SBP, systolic blood pressure.

a Missing data for some variables (20/363, 5.51%).

Supplementary Table 3. Sensitivity Analyses of Primary and Secondary Outcomes for ICH Patients by Age 

| Outcome | Unadjusted value | P Value | Adjusted Value a | P Value | Adjusted Value b | P Value |
| --- | --- | --- | --- | --- | --- | --- |
| 90-day mRS 4–6, OR (95% CI) |  |  |  |  |  |  |
| <70 years |  |  |  |  |  |  |
| Spring | 0.619 (0.325–1.177) | 0.144 | 0.559 (0.281–1.113) | 0.098 | 0.563 (0.278–1.143) | 0.112 |
| Summer | 0.556 (0.263–1.176) | 0.125 | 0.514 (0.230–1.150) | 0.105 | 0.589 (0.256–1.356) | 0.214 |
| Autumn | 0.273 (0.135–0.552) | <0.001 | 0.254 (0.121–0.534) | <0.001 | 0.335 (0.158–0.713) | 0.005 |
| Winter | Ref. | Ref. | Ref. | Ref. | Ref. | Ref. |
| ≥70 years |  |  |  |  |  |  |
| Spring | 0.731 (0.259–2.060) | 0.553 | 0.888 (0.275–2.861) | 0.842 | 1.062 (0.296–3.807) | 0.927 |
| Summer | 0.256 (0.042–1.551) | 0.138 | 0.313 (0.049–2.017) | 0.222 | 0.564 (0.080–3.973) | 0.565 |
| Autumn | 0.855 (0.252–2.898) | 0.801 | 0.868 (0.217–3.468) | 0.842 | 1.010 (0.226–4.515) | 0.990 |
| Winter | Ref. | Ref. | Ref. | Ref. | Ref. | Ref. |
| 90-day Barthel Index ≤60, OR (95% CI) |  |  |  |  |  |  |
| <70 years |  |  |  |  |  |  |
| Spring | 0.587 (0.308–1.117) | 0.105 | 0.543 (0.275–1.074) | 0.079 | 0.532 (0.259–1.093) | 0.086 |
| Summer | 0.480 (0.225–1.022) | 0.057 | 0.447 (0.200–1.002) | 0.051 | 0.533 (0.227–1.254) | 0.150 |
| Autumn | 0.344 (0.175–0.675) | 0.002 | 0.338 (0.168–0.682) | 0.002 | 0.465 (0.223–0.972) | 0.042 |
| Winter | Ref. | Ref. | Ref. | Ref. | Ref. | Ref. |
| ≥70 years |  |  |  |  |  |  |
| Spring | 0.561 (0.194–1.626) | 0.287 | 0.741 (0.226–2.425) | 0.620 | 0.645 (0.166–2.502) | 0.526 |
| Summer | 0.178 (0.029–1.093) | 0.062 | 0.217 (0.033–1.406) | 0.109 | 0.387 (0.054–2.789) | 0.346 |
| Autumn | 0.593 (0.171–2.056) | 0.410 | 0.559 (0.137–2.282) | 0.418 | 0.424 (0.081–2.219) | 0.310 |
| Winter | Ref. | Ref. | Ref. | Ref. | Ref. | Ref. |
| Prolonged length of stay, OR (95% CI) |  |  |  |  |  |  |
| <70 years |  |  |  |  |  |  |
| Spring | 0.395 (0.186–0.840) | 0.016 | 0.390 (0.180–0.844) | 0.017 | 0.342 (0.115–0.688) | 0.008 |
| Summer | 0.300 (0.130–0.693) | 0.005 | 0.311 (0.131–0.736) | 0.008 | 0.282 (0.115–0.688) | 0.005 |
| Autumn | 0.271 (0.130–0.568) | <0.001 | 0.252 (0.118–0.538) | <0.001 | 0.264 (0.120–0.580) | <0.001 |
| Winter | Ref. | Ref. | Ref. | Ref. | Ref. | Ref. |
| ≥70 years |  |  |  |  |  |  |
| Spring | 0.258 (0.082–0.815) | 0.021 | 0.238 (0.068–0.833) | 0.023 | 0.355 (0.100–1.269) | 0.111 |
| Summer | 0.179 (0.028–1.136) | 0.068 | 0.168 (0.024–1.155) | 0.070 | 0.187 (0.024–1.441) | 0.108 |
| Autumn | 0.615 (0.157–2.419) | 0.487 | 0.609 (0.146–2.547) | 0.497 | 0.570 (0.123–2.644) | 0.473 |
| Winter | Ref. | Ref. | Ref. | Ref. | Ref. | Ref. |
| Hematoma expansion, OR (95% CI) |  |  |  |  |  |  |
| <70 years |  |  |  |  |  |  |
| Spring | 0.588 (0.286–1.209) | 0.149 | 0.498 (0.227–1.093) | 0.082 | 0.530 (0.236–1.192) | 0.125 |
| Summer | 0.987 (0.452–2.154) | 0.974 | 0.980 (0.422–2.274) | 0.962 | 1.159 (0.487–2.760) | 0.739 |
| Autumn | 0.421 (0.196–0.905) | 0.027 | 0.385 (0.171–0.868) | 0.021 | 0.505 (0.219–1.168) | 0.110 |
| Winter | Ref. | Ref. | Ref. | Ref. | Ref. | Ref. |
| ≥70 years |  |  |  |  |  |  |
| Spring | 0.237 (0.077–0.731) | 0.012 | 0.209 (0.053–0.830) | 0.026 | 0.173 (0.043–0.701) | 0.014 |
| Summer | 0.131 (0.014–1.242) | 0.076 | 0.159 (0.015–1.642) | 0.123 | 0.172 (0.016–1.872) | 0.148 |
| Autumn | 0.667 (0.196–2.268) | 0.516 | 0.915 (0.215–3.904) | 0.905 | 0.855 (0.200–3.665) | 0.833 |
| Winter | Ref. | Ref. | Ref. | Ref. | Ref. | Ref. |

Abbreviations: CI, confidence interval; ICH, intracerebral hemorrhage; mRS, modified Rankin Scale; OR, odds ratio; Ref., reference category.
a Adjusted for age, sex, hypertension, diabetes mellitus, previous stroke, smoking and alcohol consumption.
b Adjusted for age, sex, systolic blood pressure, diastolic blood pressure, baseline ICH volume and ICH location.

Supplementary Table 4. Sensitivity Analyses of Primary and Secondary Outcomes for ICH Patients by Sex 

| Outcome | Unadjusted value | p Value | adjusted Value a | p Value | adjusted Value b | p Value |
| --- | --- | --- | --- | --- | --- | --- |
| 90-day mRs 4-6, OR (95% CI) |  |  |  |  |  |  |
| Male |  |  |  |  |  |  |
| spring | 0.694(0.357-1.348) | 0.281 | 0.628(0.306-1.285) | 0.203 | 0.630(0.297-1.334) | 0.227 |
| summer | 0.680(0.294-1.570) | 0.366 | 0.753(0.309-1.836) | 0.532 | 0.786(0.312-1.979) | 0.610 |
| autumn | 0.359(0.173-0.745) | 0.006 | 0.340(0.152-0.757) | 0.008 | 0.385(0.167-0.887) | 0.025 |
| winter | Ref. | Ref. | Ref. | Ref. | Ref. | Ref. |
| Female |  |  |  |  |  |  |
| spring | 0.616(0.238-1.593) | 0.317 | 0.712(0.261-1.945) | 0.508 | 0.718(0.247-2.086) | 0.542 |
| summer | 0.240(0.067-0.858) | 0.028 | 0.240(0.057-1.005) | 0.051 | 0.232(0.048-1.110) | 0.067 |
| autumn | 0.360(0.127-1.017) | 0.054 | 0.393(0.133-1.160) | 0.091 | 0.325(0.098-1.085) | 0.068 |
| winter | Ref. | Ref. | Ref. | Ref. | Ref. | Ref. |
| 90-day Barthel Index ≤60, OR (95% CI) |  |  |  |  |  |  |
| Male |  |  |  |  |  |  |
| spring | 0.608(0.312-1.184) | 0.144 | 0.533(0.259-1.098) | 0.088 | 0.512(0.237-1.107) | 0.089 |
| summer | 0.529(0.228-1.231) | 0.140 | 0.583(0.236-1.439) | 0.242 | 0.619(0.240-1.601) | 0.323 |
| autumn | 0.388(0.189-0.796) | 0.010 | 0.380(0.173-0.831) | 0.015 | 0.461(0.201-1.060) | 0.068 |
| winter | Ref. | Ref. | Ref. | Ref. | Ref. | Ref. |
| Female |  |  |  |  |  |  |
| spring | 0.630(0.244-1.623) | 0.338 | 0.722(0.268-1.948) | 0.520 | 0.709(0.238-2.116) | 0.538 |
| summer | 0.216(0.060-0.772) | 0.018 | 0.211(0.051-0.880) | 0.033 | 0.186(0.035-0.987) | 0.048 |
| autumn | 0.383(0.138-1.062) | 0.065 | 0.427(0.150-1.221) | 0.112 | 0.319(0.091-1.119) | 0.074 |
| winter | Ref. | Ref. | Ref. | Ref. | Ref. | Ref. |
| Prolonged length of stay, OR (95% CI) |  |  |  |  |  |  |
| Male |  |  |  |  |  |  |
| spring | 0.288(0.134-0.618) | 0.001 | 0.305(0.139-0.674) | 0.003 | 0.285(0.126-0.645) | 0.003 |
| summer | 0.422(0.168-1.061) | 0.067 | 0.413(0.518-1.079) | 0.071 | 0.384(0.143-1.032) | 0.058 |
| autumn | 0.359(0.162-0.795) | 0.012 | 0.311(0.135-0.716) | 0.006 | 0.344(0.145-0.814) | 0.015 |
| winter | Ref. | Ref. | Ref. | Ref. | Ref. | Ref. |
| Female |  |  |  |  |  |  |
| spring | 0.418(0.145-1.200) | 0.105 | 0.320(0.102-1.009) | 0.052 | 0.351(0.109-1.128) | 0.079 |
| summer | 0.156(0.044-0.554) | 0.004 | 0.132(0.034-0.503) | 0.003 | 0.120(0.027-0.531) | 0.005 |
| autumn | 0.286(0.097-0.841) | 0.023 | 0.262(0.084-0.820) | 0.021 | 0.223(0.068-0.738) | 0.014 |
| winter | Ref. | Ref. | Ref. | Ref. | Ref. | Ref. |
| Hematoma expansion, OR (95% CI) |  |  |  |  |  |  |
| Male |  |  |  |  |  |  |
| spring | 0.519(0.256-1.052) | 0.069 | 0.371(0.167-0.825) | 0.015 | 0.417(0.186-0.936) | 0.034 |
| summer | 0.751(0.316-1.784) | 0.517 | 0.639(0.247-1.651) | 0.355 | 0.776(0.300-2.010) | 0.602 |
| autumn | 0.347(0.157-0.765) | 0.009 | 0.278(0.112-0.687) | 0.006 | 0.357(0.144-0.888) | 0.027 |
| winter | Ref. | Ref. | Ref. | Ref. | Ref. | Ref. |
| Female |  |  |  |  |  |  |
| spring | 0.224(0.057-0.883) | 0.033 | 0.293(0.069-1.242) | 0.096 | 0.344(0.079-1.494) | 0.154 |
| summer | 0.578(0.158-2.115) | 0.407 | 0.766(0.191-3.066) | 0.707 | 0.911(0.209-3.960) | 0.901 |
| autumn | 0.867(0.298-2.521) | 0.793 | 1.033(0.331-3.219) | 0.956 | 1.238(0.376-4.083) | 0.725 |
| winter | Ref. | Ref. | Ref. | Ref. | Ref. | Ref. |

Abbreviations: CI, confidence interval; ICH, intracerebral hemorrhage; mRS, modified Rankin Scale; OR, odds ratio; Ref., reference category.
a Adjusted for age, sex, hypertension, diabetes mellitus, previous stroke, smoking and alcohol consumption.
b Adjusted for age, sex, systolic blood pressure, diastolic blood pressure, baseline ICH volume and ICH location.

Supplementary Table 5. Sensitivity Analyses of Primary and Secondary Outcomes for ICH Patients with GCS ≥ 5

| Outcome | Unadjusted OR (95% CI) | P Value | Adjusted ORa (95% CI) | P Value | Adjusted ORb (95% CI) | P Value |
| --- | --- | --- | --- | --- | --- | --- |
| 90-day mRs 4-6, OR (95% CI) |  |  |  |  |  |  |
| Spring | 0.697(0.391-1.242) | 0.221 | 0.668(0.368-1.212) | 0.185 | 0.614(0.330-1.144) | 0.124 |
| Summer | 0.372(0.167-0.829) | 0.016 | 0.394(0.173-0.896) | 0.026 | 0.369(0.154-0.882) | 0.025 |
| Autumn | 0.360(0.189-0.689) | 0.002 | 0.373(0.192-0.724) | 0.004 | 0.388(0.194-0.776) | 0.007 |
| Winter | Ref. | Ref. | Ref. | Ref. | Ref. | Ref. |
| 90-day Barthel Index ≤ 60, OR (95% CI) |  |  |  |  |  |  |
| Spring | 0.631(0.355-1.122) | 0.117 | 0.601(0.331-1.090) | 0.094 | 0.551(0.293-1.037) | 0.065 |
| Summer | 0.286(0.126-0.649) | 0.003 | 0.302(0.130-0.701) | 0.005 | 0.284(0.115-0.703) | 0.006 |
| Autumn | 0.397(0.212-0.743) | 0.004 | 0.414(0.217-0.790) | 0.007 | 0.456(0.230-0.905) | 0.025 |
| Winter | Ref. | Ref. | Ref. | Ref. | Ref. | Ref. |
| Prolonged length of stay, OR (95% CI) |  |  |  |  |  |  |
| Spring | 0.348(0.187-0.649) | <0.001 | 0.359(0.190-0.681) | 0.002 | 0.334(0.172-0.648) | 0.001 |
| Summer | 0.309(0.144-0.663) | 0.003 | 0.306(0.139-0.673) | 0.003 | 0.284(0.125-0.646) | 0.003 |
| Autumn | 0.354(0.186-0.675) | 0.002 | 0.311(0.159-0.607) | <0.001 | 0.326(0.163-0.652) | 0.002 |
| Winter | Ref. | Ref. | Ref. | Ref. | Ref. | Ref. |
| Hematoma expansion, OR (95% CI) |  |  |  |  |  |  |
| Spring | 0.452(0.231-0.885) | 0.020 | 0.393(0.197-0.786) | 0.008 | 0.393(0.193-0.798) | 0.010 |
| Summer | 0.670(0.296-1.515) | 0.336 | 0.642(0.276-1.498) | 0.306 | 0.739(0.313-1.744) | 0.490 |
| Autumn | 0.533(0.268-1.059) | 0.072 | 0.541(0.267-1.094) | 0.087 | 0.626(0.305-1.287) | 0.202 |
| Winter | Ref. | Ref. | Ref. | Ref. | Ref. | Ref. |

Abbreviations: CI, confidence interval; ICH, intracerebral hemorrhage; mRS, modified Rankin Scale; OR, odds ratio; Ref., reference category.
a Adjusted for age, sex, hypertension, diabetes mellitus, previous stroke, smoking and alcohol consumption.
b Adjusted for age, sex, systolic blood pressure, diastolic blood pressure, baseline ICH volume and ICH location.

Supplementary Table 6. Sensitivity Analyses of Primary and Secondary Outcomes for ICH Patients with GCS ≥ 13

| Outcome | Unadjusted OR (95% CI) | P Value | Adjusted ORa (95% CI) | P Value | Adjusted ORb (95% CI) | P Value |
| --- | --- | --- | --- | --- | --- | --- |
| 90-day mRs 4-6, OR (95% CI) |  |  |  |  |  |  |
| Spring | 0.699(0.308-1.586) | 0.391 | 0.617(0.258-1.474) | 0.277 | 0.579(0.240-1.398) | 0.224 |
| Summer | 0.371(0.122-1.125) | 0.080 | 0.365(0.116-1.148) | 0.085 | 0.379(0.119-1.205) | 0.100 |
| Autumn | 0.458(0.192-1.095) | 0.079 | 0.452(0.180-1.134) | 0.091 | 0.423(0.163-1.095) | 0.076 |
| Winter | Ref. | Ref. | Ref. | Ref. | Ref. | Ref. |
| 90-day Barthel Index ≤ 60, OR (95% CI) |  |  |  |  |  |  |
| Spring | 0.696(0.312-1.557) | 0.378 | 0.646(0.275-1.522) | 0.318 | 0.602(0.249-1.458) | 0.261 |
| Summer | 0.264(0.081-0.864) | 0.028 | 0.277(0.082-0.937) | 0.039 | 0.283(0.082-0.973) | 0.045 |
| Autumn | 0.564(0.248-1.283) | 0.172 | 0.628(0.264-1.496) | 0.294 | 0.622(0.248-1.559) | 0.311 |
| Winter | Ref. | Ref. | Ref. | Ref. | Ref. | Ref. |
| Prolonged length of stay, OR (95% CI) |  |  |  |  |  |  |
| Spring | 0.206(0.093-0.457) | <0.001 | 0.174(0.075-0.403) | <0.001 | 0.150(0.062-0.362) | <0.001 |
| Summer | 0.270(0.110-0.662) | 0.004 | 0.254(0.101-0.643) | 0.004 | 0.221(0.081-0.598) | 0.003 |
| Autumn | 0.236(0.107-0.518) | <0.001 | 0.167(0.072-0.389) | <0.001 | 0.165(0.067-0.404) | <0.001 |
| Winter | Ref. | Ref. | Ref. | Ref. | Ref. | Ref. |
| Hematoma expansion, OR (95% CI) |  |  |  |  |  |  |
| Spring | 0.391(0.134-1.139) | 0.085 | 0.299(0.097-0.919) | 0.035 | 0.355(0.118-1.071) | 0.066 |
| Summer | 1.256(0.462-3.420) | 0.655 | 1.387(0.481-3.999) | 0.545 | 1.447(0.508-4.125) | 0.489 |
| Autumn | 0.754(0.299-1.902) | 0.550 | 0.826(0.312-2.186) | 0.700 | 0.891(0.332-2.388) | 0.818 |
| Winter | Ref. | Ref. | Ref. | Ref. | Ref. | Ref. |

Abbreviations: CI, confidence interval; ICH, intracerebral hemorrhage; mRS, modified Rankin Scale; OR, odds ratio; Ref., reference category.
a Adjusted for age, sex, hypertension, diabetes mellitus, previous stroke, smoking and alcohol consumption.
b Adjusted for age, sex, systolic blood pressure, diastolic blood pressure, baseline ICH volume and ICH location.
